# A Note on the Distribution of Cancer in Some Endogamous Groups in Western India

**DOI:** 10.1038/bjc.1971.77

**Published:** 1971-12

**Authors:** K. Jayant, V. Balakrishnan, L. D. Sanghvi

## Abstract

Endogamous groups in Western India have been known to have a wide degree of genetic diversity. Analysis of the data available on some of the endogamous groups belonging to two main communities attending the Tata Memorial Hospital, Bombay, viz. Hindus from Maharashtra, and Hindus from Gujarat show that one of the endogamous groups, the Maharashtrian Brahmin has a significantly different pattern of cancer site distribution compared to the other groups studied. Maharashtrian Brahmins have a low relative frequency of cancer of the oral cavity and high relative frequencies of cancer of the oropharynx and cancer of the oesophagus. The tobacco chewing habit pattern seems to have a bearing on the proportion of cancer of the oral cavity observed in the groups studied. However, in the case of cancer of the oropharynx and cancer of the oesophagus, factors besides smoking and chewing habits seem to be playing a role. A further study is indicated to clarify the points raised by the study.


### A NOTE ON THE DISTRIBUTION OF CANCER IN SOME ENDOGAMOUS GROUPS IN WESTERN INDIA

K. JAYANT, V. BALAKRISHNAN AND L. D. SANGHVI

*From the Epidemiology Division, Cancer Research Institute, Tata Memorial Centre, Bombay-12, India*

Received for publication July 21, 1971

**SUMMARY.—Endogamous groups in Western India have been known to have a wide degree of genetic diversity. Analysis of the data available on some of the endogamous groups belonging to two main communities attending the Tata Memorial Hospital, Bombay, viz. Hindus from Maharashtra, and Hindus from Gujarat show that one of the endogamous groups, the Maharashtrian Brahmin has a significantly different pattern of cancer site distribution compared to the other groups studied. Maharashtrian Brahmins have a low relative frequency of cancer of the oral cavity and high relative frequencies of cancer of the oropharynx and cancer of the oesophagus. The tobacco chewing habit pattern seems to have a bearing on the proportion of cancer of the oral cavity observed in the groups studied. However, in the case of cancer of the oropharynx and cancer of the oesophagus, factors besides smoking and chewing habits seem to be playing a role. A further study is indicated to clarify the points raised by the study.**

EARLIER studies from India (Khanolkar, 1959; Paymaster, 1967) demonstrated a great deal of variation in the organ distribution of cancer in different religious communities and in different linguistic groups. Social institution of caste which has prevailed in the country for more than two millenniums, has not yet been used to explain variation in cancer distribution. It has, however, been shown through a number of detailed studies in recent years that there is a wide degree of genetic diversity among these castes (Sanghvi, 1966) and the extent of the intermarriage between different castes even today appears to be less than 1% (Ali, 1968). In view of this, it occurred to us that a study of cancer distribution in different endogamous groups would be of interest.

### MATERIAL

Data from the Tata Memorial Hospital for the 5 year period 1946–50 when the caste of the patient was usually recorded have been analysed. Relative frequency ratios for the caste groups under study have been worked out for various cancer sites.

Hindus are composed of a large number of endogamous groups commonly known as castes. In Maharashtra one of the endogamous groups, the Marathas, who are mainly agriculturists, form 40% of the Hindus and so the data on Marathas were large enough for considering them as a separate group. But various subgroups or occupational castes, Brahmins and Harijans, could not be considered separately due to paucity of data. Further, some groups like tribals had to be excluded from any detailed study.

Among the Hindus from Gujarat, occupational castes, agricultural castes, Lohanas, trading castes and Brahmins have been considered (see Appendix).

Patterns of oral and pharyngeal cancers, cancer of the oesophagus and in the females, cancer of the breast and cervix are studied. The other sites of cancer are grouped together. For oral and pharyngeal cancers, classification given by Paymaster (1962) has been followed.

TABLE I.—*Distribution of Cancer at Different Sites in Some Groups of Hindus from Maharashtra*

Males

| | Harijans | | Marathas | | Occupational castes | | Brahmins | | All Hindus from Maharashtra | |
|---|---|---|---|---|---|---|---|---|---|---|
| | No. | % | No. | % | No. | % | No. | % | No. | % |
| Ca. Oral cavity . . . | 37 | 33·33 | 124 | 26·38 | 56 | 20·22 | 36 | 11·65 | 343 | 21·64 |
| Ca. Oropharynx . . . | 20 | 18·02 | 87 | 18·51 | 69 | 24·91 | 79 | 25·57 | 347 | 21·89 |
| Ca. Hypopharynx . . . | 17 | 15·32 | 87 | 18·51 | 52 | 18·77 | 40 | 12·94 | 275 | 17·35 |
| Ca. Nasopharynx . . . | — | — | 2 | 0·43 | 1 | 0·36 | 1 | 0·32 | 5 | 0·32 |
| Oral + pharyngeal carcinomas . . . | 74 | 66·67 | 300 | 63·83 | 178 | 64·26 | 156 | 50·48 | 970 | 61·20 |
| Ca. Oesophagus . . . | 5 | 4·50 | 32 | 6·81 | 22 | 7·94 | 66 | 21·36 | 175 | 11·04 |
| Other carcinomas . . . | 32 | 28·82 | 138 | 29·36 | 77 | 27·80 | 87 | 28·15 | 440 | 27·76 |
| Total carcinomas | 111 | | 470 | | 277 | | 309 | | 1585 | |

Females

| | Harijans | | Marathas | | Occupational castes | | Brahmins | | All Hindus from Maharashtra | |
|---|---|---|---|---|---|---|---|---|---|---|
| | No. | % | No. | % | No. | % | No. | % | No. | % |
| Ca. Oral cavity . . . | 24 | 22·22 | 68 | 17·99 | 35 | 16·35 | 11 | 4·25 | 172 | 14·05 |
| Ca. Oropharynx . . . | 2 | 1·85 | 15 | 3·97 | 10 | 4·67 | 6 | 2·32 | 46 | 3·76 |
| Ca. Hypopharynx . . . | 2 | 1·85 | 11 | 2·91 | 6 | 2·80 | 5 | 1·93 | 33 | 2·70 |
| Oral + pharyngeal carcinomas . . . | 28 | 25·92 | 94 | 24·87 | 51 | 23·83 | 22 | 8·49 | 251 | 20·51 |
| Ca. Oesophagus . . . | 1 | 0·92 | 19 | 5·03 | 16 | 7·48 | 19 | 7·33 | 62 | 5·06 |
| Ca. Cervix . . . | 62 | 57·41 | 197 | 52·12 | 102 | 47·66 | 132 | 50·96 | 631 | 51·55 |
| Ca. Breast . . . | 5 | 4·63 | 38 | 10·05 | 20 | 9·34 | 49 | 18·92 | 146 | 11·93 |
| Other carcinomas . . . | 12 | 11·11 | 30 | 7·94 | 25 | 11·68 | 37 | 14·28 | 134 | 10·95 |
| Total carcinomas . . | 108 | | 378 | | 214 | | 259 | | 1224 | |

### RESULTS

Among the Hindus from Maharashtra the oral and pharyngeal cancer proportion to the total carcinomas is 61·20% for males and 20·51% for females. These are of the same order as those reported (*viz.* 58·7% and 19·0% respectively) for a larger series from the same hospital (Paymaster, 1967). However, when the caste groups are considered separately, Brahmins show a comparatively low proportion of oral and pharyngeal cancers, 50·48% in the males and 8·49% in the females. The other caste groups considered are not markedly different from each other.

The corresponding proportion among all Gujaratis in the study is 72·86% for males and 18·98% for females which compare well with those reported earlier from the same hospital, *viz.* 68·7% and 18·0% respectively (Paymaster, 1967).

FIG. 1.—Distribution of carcinoma in different sites in some groups from Maharashtra.

FIG. 2.—Distribution of carcinoma in different sites in some groups from Gujarat.

The proportion of oral and pharyngeal cancer to total carcinomas varies from 68·49% among Brahmins to 81·91% among the Lohanas. In the females the variation is great among the caste groups probably due to small numbers.

Fig. 1 and 2 and Tables I and II give the percentage distribution of patients with carcinomas of oral cavity, oropharynx, hypopharynx, oesophagus and carcinomas for other sites for males as also of cervix and breast for females, among the groups under consideration.

The Maharashtrian Brahmin males show the lowest proportion of cancer of the oral cavity compared to the other caste groups and Harijans show the highest. The reverse is observed for cancer of the oesophagus in that the Brahmin males show the highest proportion of cancer of the oesophagus, *viz.* 21·36% compared to the other caste groups which show proportions varying from 4·50% to 7·94%. Brahmin males have a significantly different pattern of cancer site distribution from Harijan males ($\chi^2 = 38{\cdot}09$, $P < 0{\cdot}001$), Maratha males ($\chi^2 = 59{\cdot}11$, $P < 0{\cdot}001$) or males belonging to occupational castes ($\chi^2 = 27{\cdot}32$, $P < 0{\cdot}001$). Harijans and Marathas have similar patterns whereas the group of occupational castes has a pattern which differs from those of Marathas or Harijans though not significantly.

TABLE II.—*Distribution of Cancer at Different Sites in Some Groups of Hindus from Gujarat*

| | Males | | | | | | | | | | | |
|---|---|---|---|---|---|---|---|---|---|---|---|---|
| | Occupational castes | | Agricultural castes | | Lohanas | | Trading castes | | Brahmins | | All Hindus from Gujarat | |
| | No. | % | No. | % | No. | % | No. | % | No. | % | No. | % |
| Ca. Oral cavity | 22 | 6·36 | 16 | 6·90 | 11 | 10·48 | 43 | 7·41 | 18 | 5·79 | 162 | 7·88 |
| Ca. Oropharynx | 183 | 52·89 | 114 | 49·14 | 54 | 51·43 | 265 | 45·69 | 143 | 45·98 | 957 | 46·55 |
| Ca. Hypopharynx | 73 | 21·10 | 43 | 18·53 | 20 | 19·05 | 103 | 17·76 | 50 | 16·08 | 366 | 17·80 |
| Ca. Nasopharynx | 2 | 0·58 | 1 | 0·43 | 1 | 0·95 | 2 | 0·34 | 2 | 0·64 | 13 | 0·63 |
| Oral + pharyngeal carcinoma | 280 | 80·92 | 174 | 75·00 | 86 | 81·91 | 413 | 71·20 | 213 | 68·49 | 1498 | 72·86 |
| Ca. Oesophagus | 21 | 6·07 | 24 | 10·34 | 11 | 10·48 | 77 | 13·27 | 43 | 13·83 | 234 | 11·38 |
| Other carcinomas | 45 | 13·00 | 34 | 14·66 | 8 | 7·62 | 90 | 15·52 | 55 | 17·68 | 324 | 15·76 |
| Total carcinomas | 346 | | 232 | | 105 | | 580 | | 311 | | 2056 | |

Even among the females when the distribution of cancer of oral and pharyngeal regions, breast, cervix and the rest are compared, the Brahmin females have a pattern significantly different from Harijan females ($\chi^2 = 31{\cdot}87$, $P < 0{\cdot}001$), Maratha females ($\chi^2 = 38{\cdot}19$, $P < 0{\cdot}001$) or females belonging to the occupational castes ($\chi^2 = 25{\cdot}70$, $P < 0{\cdot}001$). The Brahmin females have a higher proportion of cancer of the breast compared to the other groups and like the Brahmin males a low proportion of oral and pharyngeal cancers and a high proportion of cancer of the oesophagus, compared to Harijan females though not compared to females belonging to occupational castes.

The males of the Gujarati caste groups do not show much variability except that the occupational castes differ from trading castes ($\chi^2 = 15{\cdot}89$, $P < 0{\cdot}01$) and Brahmins ($\chi^2 = 16{\cdot}28$, $P < 0{\cdot}01$). The Gujarati Brahmin males and the trading caste males have a higher proportion of cancer of the oesophagus compared to the other groups, just as Maharashtrian Brahmin males have, though the difference is not as marked as in the Maharashtrian Brahmin males.

Data on Gujarati females are not adequate for a detailed study.

### DISCUSSION

As seen above the Maharashtrian Brahmin males and females show a low relative frequency of cancer of the oral cavity quite unlike the other caste groups of Maharashtra. The Gujarati Brahmin males do not show any marked difference in the relative frequency of cancer of the oral cavity compared to the other Gujarati groups. It is likely that this is so, because the relative frequency of cancer of the oral cavity itself is known to be low among the Gujaratis (Khanolkar, 1959). It is of interest to note that as early as 1923, it was observed that among the patients seen at Neyyoor Hospital, South India, the Brahmins rarely had cancer of the buccal mucosa (Davidson, 1923). He raised the question as to whether there is any difference in the contents of the " quid " in this caste group compared to the others. Various other workers (Sanghvi *et al.*, 1955; Hirayama, 1966) have also shown the association of smoking and chewing habits to oral and pharyngeal cancers. It is possible that the variation in the relative frequencies of oral and pharyngeal cancers in the groups studied here is mainly due to the variation in the smoking and the chewing habits. As we did not have the necessary data on habits of the cancer patients themselves, we made use of data available with us on the males in the general rural population of Maharashtra. We had no such data on the Gujarati males. So our further discussion is confined to Maharashtrian males only.

TABLE III.—*Percentage Distribution of Chewing Habit*

| | Percentage with tobacco chewing habit (irrespective of the smoking habit) | Percentage addicted to tobacco chewing habit before 18 years |
|---|---|---|
| Harijans | 52·45 | 38·16 |
| Marathas | 45·49 | 29·15 |
| Occupational castes | 30·47 | 25·60 |
| Brahmins | 31·83 | 8·08 |

Smoking and chewing habits of males above 35 years of age were collected in different parts of Maharashtra during 1952. In these data, smoking and chewing habits of caste groups mentioned above were observed. However, as the habit data are not on the study group itself, our conclusions would be of a general nature and would not be supported by critical statistical analysis.

In each group the percentage of persons with the habit of tobacco chewing and the percentage of those addicted before the age of 18 years are given in Table III.

Case control studies in India and Ceylon have shown that the relative risks of cancer of the buccal mucosa and cancer of the lip among tobacco chewers are as high as 7 and 5 respectively, assuming the risk among non-chewers as 1 (Hirayama, 1966). Tobacco chewers also have a significantly higher risk of cancer of some of the other parts of the oral cavity. In Mainpuri, India, the prevalence rate of oral cancer in daily tobacco chewers was noted to be significantly higher while that for non-chewers was significantly lower than the average and tobacco chewers who had started the habit at younger ages had a higher risk of developing oral cancer (Wahi, 1968).

From the pattern observed in Table III and on the basis of known associations outlined above, one would expect a high proportion of cancer of the oral cavity in Harijans and Marathas as compared with the other two groups; also occupational castes would be expected to have a higher proportion of cancer of the oral cavity compared to Brahmins, even though both the groups have similar proportions of persons addicted to the chewing habit, in view of the marked difference in the proportions addicted before 18 years in these two groups. In our data, as we have already seen, Harijans and Marathas have the highest proportion of cancer of the oral cavity, occupational castes come next and last of all the Brahmins. It appears that the observed variation in the relative frequency of oral cancer in the castes can be ascribed to the variation in the habit of tobacco chewing (Fig. 3).

FIG. 3.—Percentage with carcinoma of the oral cavity and the tobacco chewing habit in the four groups.

TABLE IV.—*Percentage Distribution of Smoking Habit*

| | Percentage smoking bidis (irrespective of chewing) | Percentage addicted before 18 years |
|---|---|---|
| Harijans | 26·47 | 34·69 |
| Marathas | 28·34 | 26·30 |
| Occupational castes | 42·47 | 34·07 |
| Brahmins | 25·72 | 18·19 |

Table IV gives the percentages of persons addicted to the bidi smoking habit and the percentages of those addicted before 18 years in the four groups.

The habit of smoking bidis is shown to be significantly associated with cancer of the oropharynx (Sanghvi *et al.*, 1955). The relative risk of smokers getting cancer of the base of the tongue has been shown to be 4 and that of the rest of the oropharynx to be 5, assuming the risk of non-smokers as 1 (Hirayama, 1966). There are no critical reports about the aspect of age of addiction to bidi smoking and pharyngeal cancers.

In our data (Table IV) occupational castes have the highest proportion addicted to smoking and so a high proportion of cancer of the oropharynx could be expected in this group; Brahmins who have the smallest proportion addicted would be expected to have low proportions of these types of cancers. In our study group, quite contrary to expectation, occupational castes and Brahmins have similar proportions of cancer of the oropharynx. This finding cannot be explained even if we assume a dose effect of bidi smoking due to different proportions being addicted at younger ages. As seen in Table IV, occupational castes not only have a high proportion addicted to smoking but they also have a high proportion addicted at younger ages. We further considered the distribution of the components of oropharynx, in particular, that of cancer of the base of the tongue, where the combined habit of smoking and chewing of tobacco has been implicated (Sanghvi *et al.*, 1955). Again, Brahmins, who have the smallest proportion of persons addicted to the combined habit compared to the other groups, have the highest proportion of cancer of the base of the tongue. These observations seem to

FIG. 4.—Percentage with carcinoma of the oropharynx and oesophagus, and the bidi smoking habit in the four groups.

indicate that other factors besides smoking and chewing habits are responsible for the high proportion of cancer of the oropharynx in the Brahmins (Fig. 4).

In the case of cancer of the oesophagus, smoking has been implicated by many workers. The habit of bidi smoking was found to be significantly associated with cancer of the oesophagus (Sanghvi *et al.*, 1955) and the risk has been shown to increase with the number of cigarettes smoked (Wynder and Bross, 1961).

In our data, Brahmins have the highest proportion of cancer of the oesophagus even though they are not the ones with the highest proportion addicted to smoking (Fig. 4). The other factor implicated in cancer of the oesophagus is alcohol. It seems unlikely that Brahmins have a higher proportion addicted to drinking compared to the others, even though we do not have any data on this factor. Smoking has been found to be significantly associated with upper two-thirds of the oesophagus but not in cancer of the lower third (Paymaster *et al.*, 1968). Our data are not sufficiently large to study this aspect in detail. Irrespective of whether it is the upper two-thirds or the lower third which contributes to the high proportion of cancer of the oesophagus in Brahmins, the high relative frequency of cancer of the oesophagus calls for a detailed study on larger data.

An interesting feature in the present data is that when the proportion of cancer of the upper alimentary canal to total cancer is considered, the four groups under study do not show any significant differences.

We wish to point out that the results have to be interpreted with caution. Firstly, we have analysed relative frequencies from hospital data. (Relative frequency for a site may be high in a group either because the site in question has in fact a high incidence in the group or it may be so even with a low incidence if the total incidence of cancer in the group in question is low.) Secondly, due to paucity of data, we did not restrict ourselves to only histologically proved cases. Thirdly, the habit data are not on the diseased group itself. In the circumstances, we have merely tried to see in a general way whether the observed variations could be explained by the associations already put forward by various workers. The present findings are interesting pointers to further work.

We are deeply grateful to Dr. J. C. Paymaster, Director of the Tata Memorial Centre, and Dr. D. R. Meher-Homji, Superintendent and Chief Surgeon of the Tata Memorial Hospital, Bombay, for their permission to analyse the data. Thanks are due to the staff of the Department of Medical Records and Statistics for their co-operation.

### REFERENCES

Ali, S. G. M.—(1968) *Acta genet. Statist. med.*, **18**, 369.
Davidson, J.—(1923) *Br. med. J.*, ii, 733.
Hirayama, J.—(1966) *Bull. Wld Hlth Org.*, **34**, 41.
Khanolkar, V. R.—(1959) *Acta Un. int. Cancr.*, **15**, 67.
Paymaster, J. C.—(1962) *Cancer, N.Y.*, **15**, 578.—(1967) ‘Epidemiologic study of cancer in Western India’, in ‘Progress in Clinical Cancer’, edited by Irwing Ariel. New York (Grune & Stratton), Vol. III, p. 107.
Paymaster, J. C., Sanghvi, L. D. and Gangadharan, P.—(1968) *Cancer, N.Y.*, **21**, 279.
Sanghvi, L. D.—(1966) ‘Genetic adaptation in man’, in ‘The Biology of Human Adaptability’, edited by Paul J. Baker and J. S. Weiner. Oxford (Clarendon Press), p. 305.
Sanghvi, L. D., Rao, K. C. M. and Khanolkar, V. R.—(1955) *Br. med. J.*, i, 1111.
Wahi, P. N.—(1968) *Bull. Wld Hlth Org.*, **38**, 496.
Wynder, S. L. and Bross, I. J.—(1961) *Cancer, N.Y.*, **14**, 389.

### APPENDIX

*Agricultural castes* are castes whose main occupation is cultivation.

*Brahmins* were originally priests. There are sub-groups of them but, in our data, information on sub-groups is not available.

*Harijans* were exterior castes of old. A large number of them call themselves Harijans and do not give information on their actual sub-group.

*Lohanas* are a trading community. They have been considered separately.

*Marathas* form a distinct endogamous group and in our data the group was large enough to be considered separately. They are mainly agriculturists.

*Occupational castes* consist of various groups (of artisans) like the Bhandare (Toddy Tapper), Carpenter, etc. They were grouped together mainly because of paucity of material.

*The following is a list of the population according to castes:*

*Hindu Maharashtrian Males*

| | |
|---|---|
| Brahmins: | |
| Gaud Saraswat Brahmin | 45 |
| Other/unspecified | 264 |
| | 309 |
| Harijans: | |
| Chamar | 21 |
| Mahar | 19 |
| Other/unspecified | 71 |
| | 111 |
| Marathas: | 470 |
| Occupational castes: | |
| Bhandare | 44 |
| Koli | 34 |
| Mali | 29 |
| Agri | 27 |
| Sonar | 24 |
| Shimpi | 21 |
| Nhavi | 16 |
| Teli | 12 |
| Sutar | 12 |
| Koshti | 12 |
| Others | 46 |
| Total | 277 |

*Hindu Gujarati Males*

| | |
|---|---|
| Agricultural castes: | |
| Kanbhi | 144 |
| Other | 88 |
| | 232 |
| Brahmins: | |
| Mostly unspecified | 311 |
| | 311 |
| Lohanas: | 105 |
| Occupational castes: | |
| Mistry | 82 |
| Soni | 51 |
| Kharvi | 35 |
| Darji | 34 |
| Lohar | 30 |
| Kumbhar | 19 |
| Kansara | 14 |
| Others | 81 |
| Total | 346 |
| Trading castes: | |
| Bania | 377 |
| Jain Bania | 167 |
| Others | 36 |
| Total | 580 |


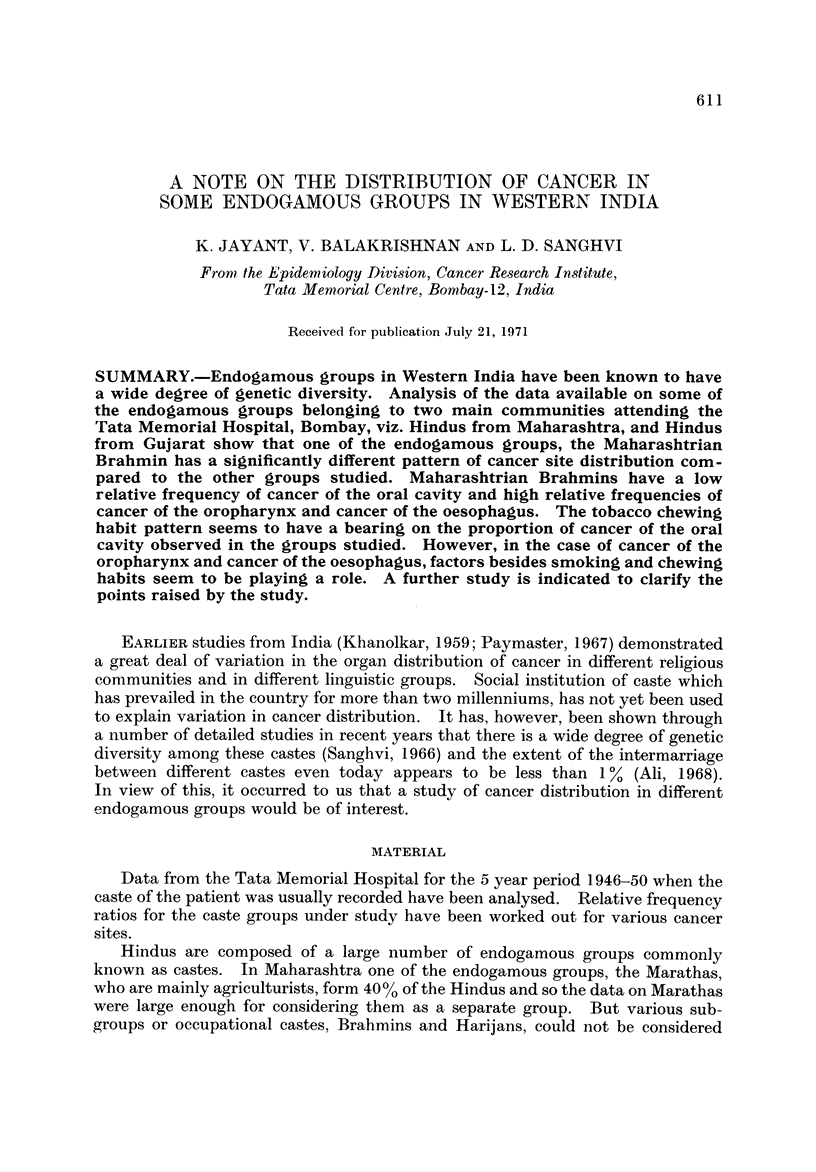

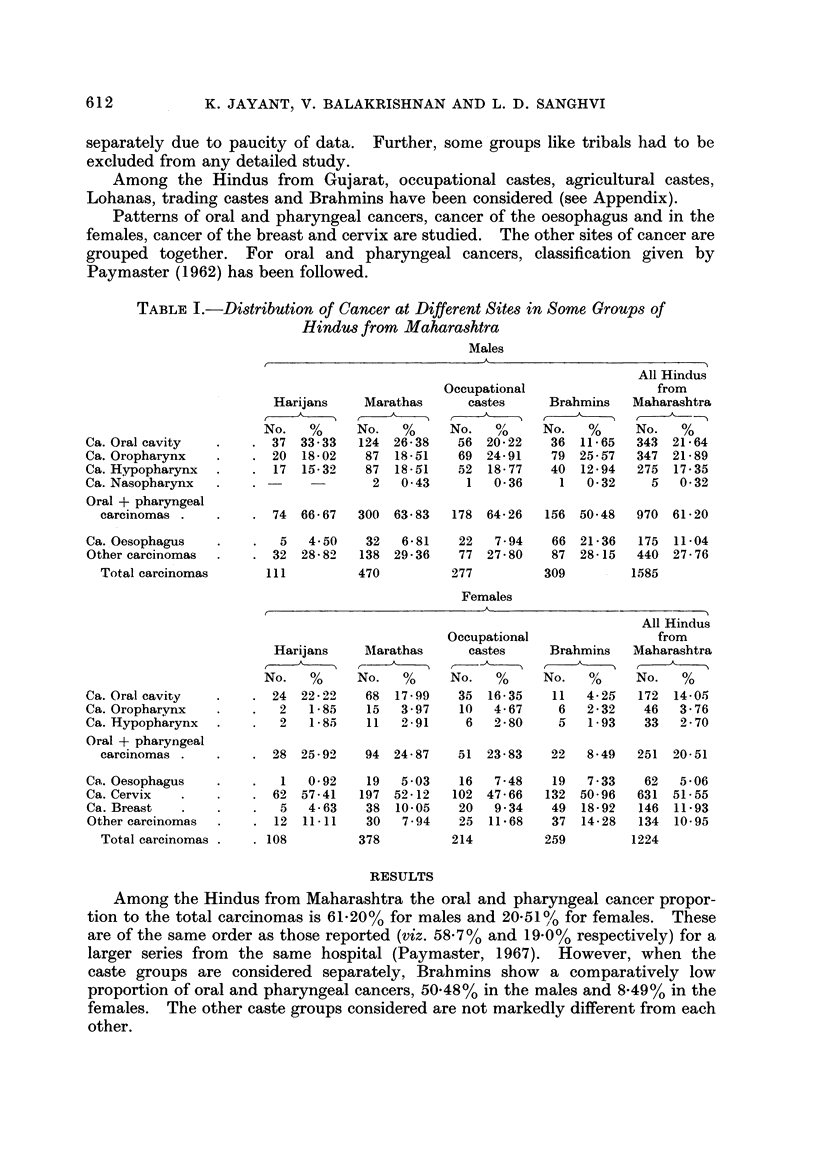

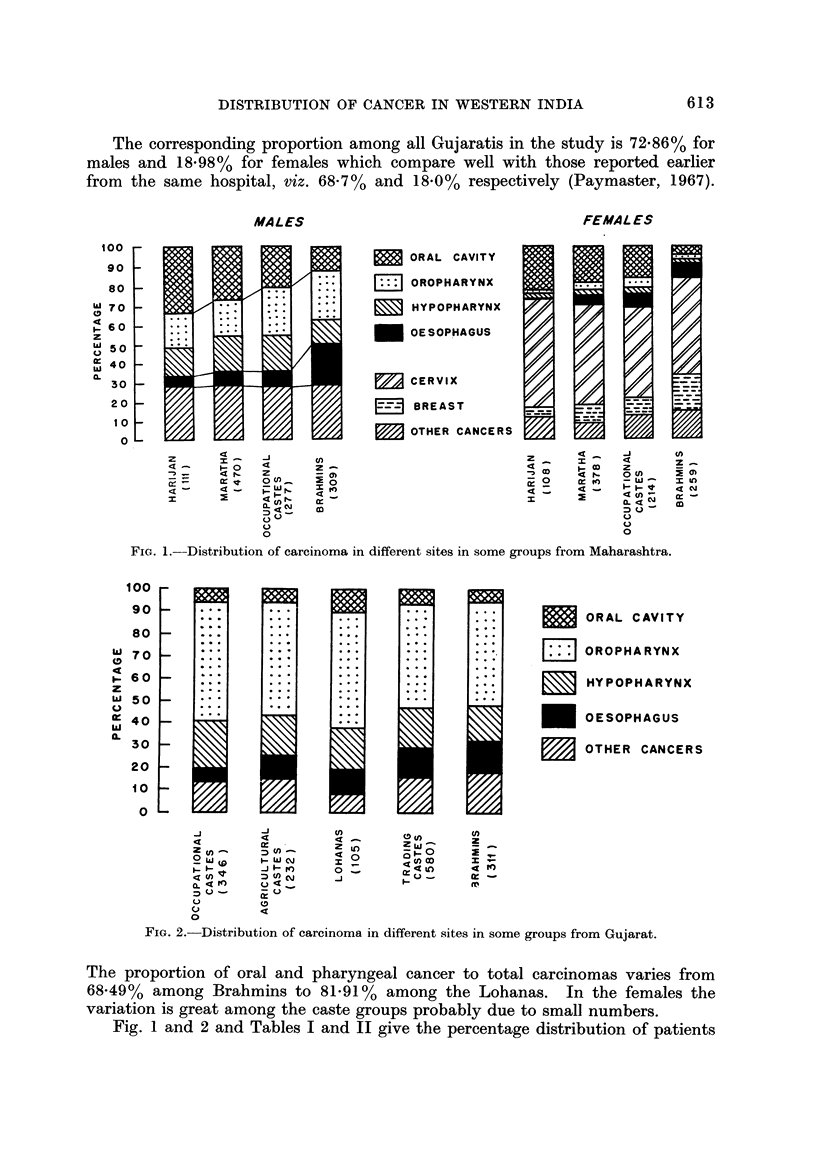

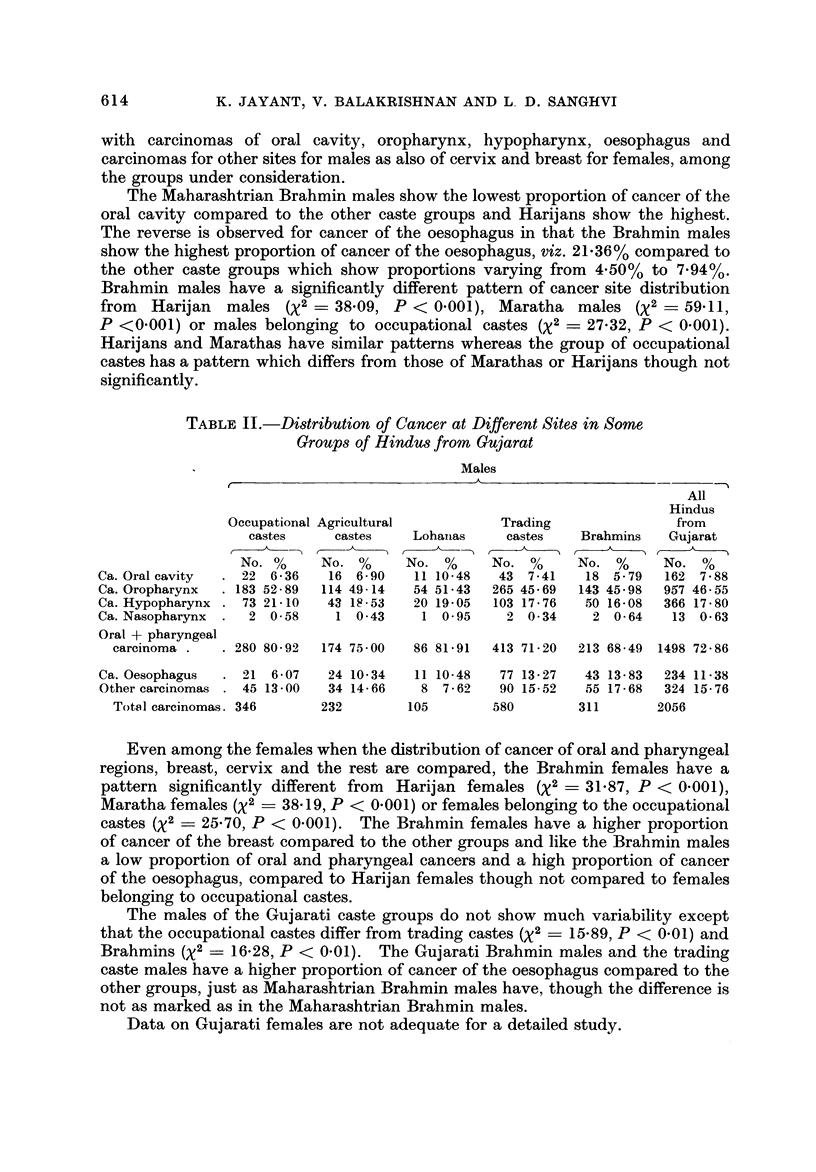

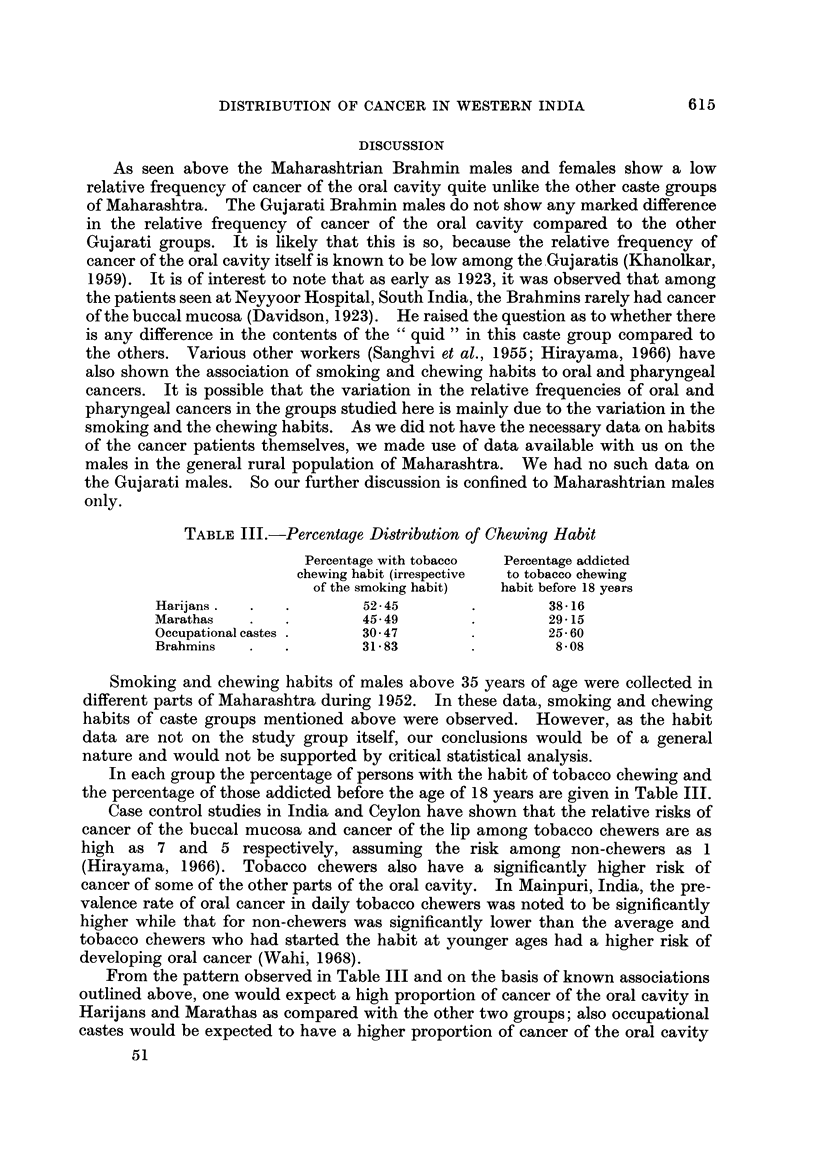

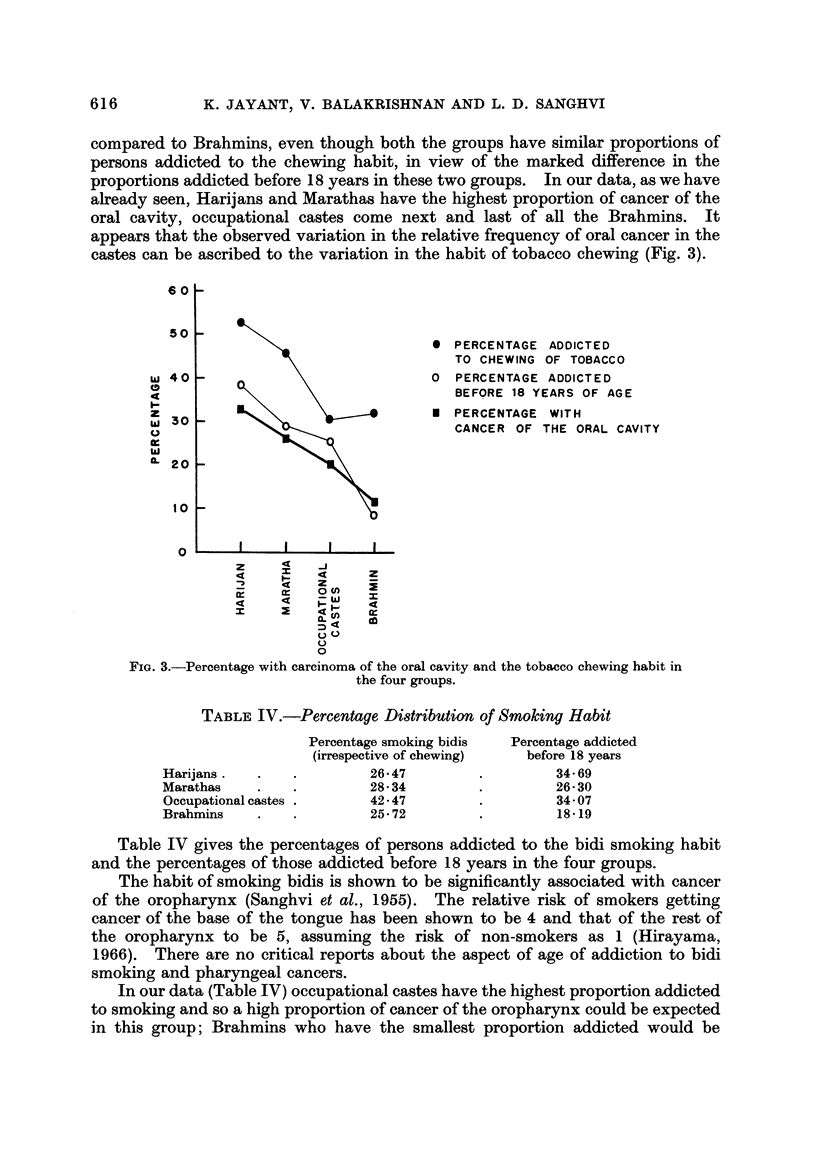

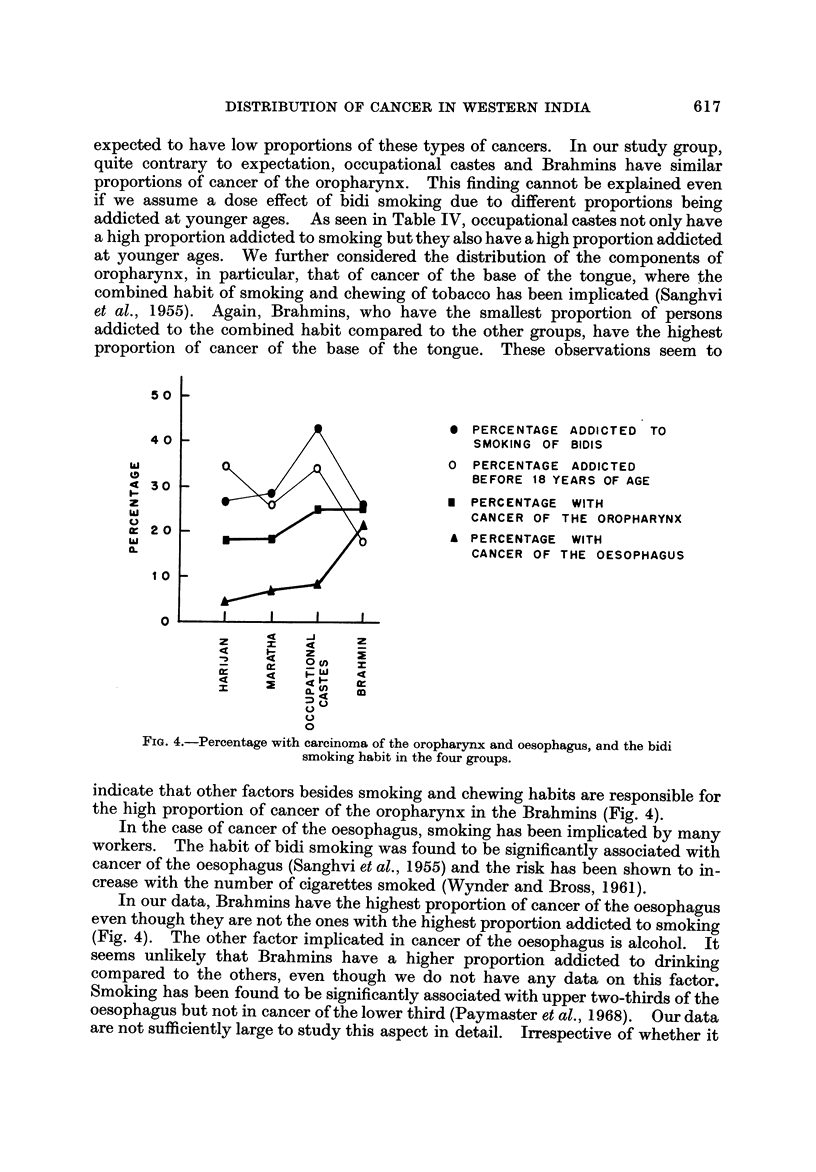

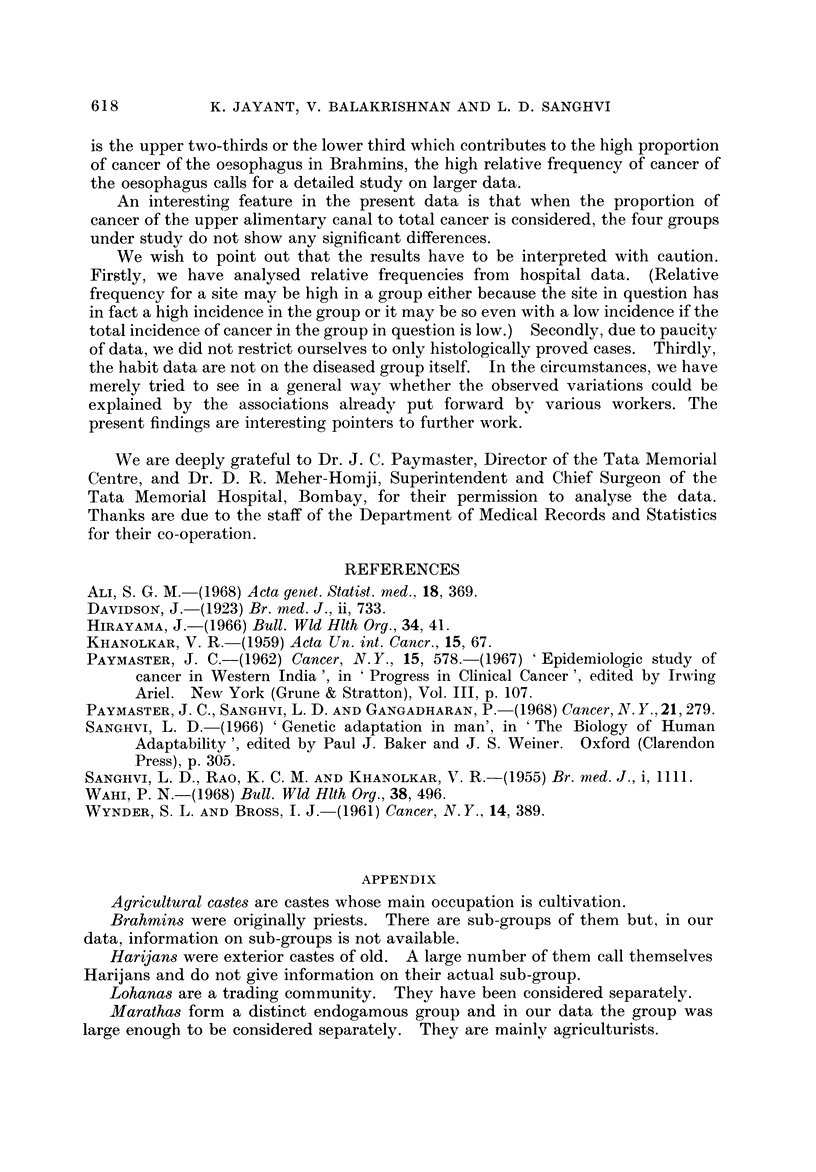

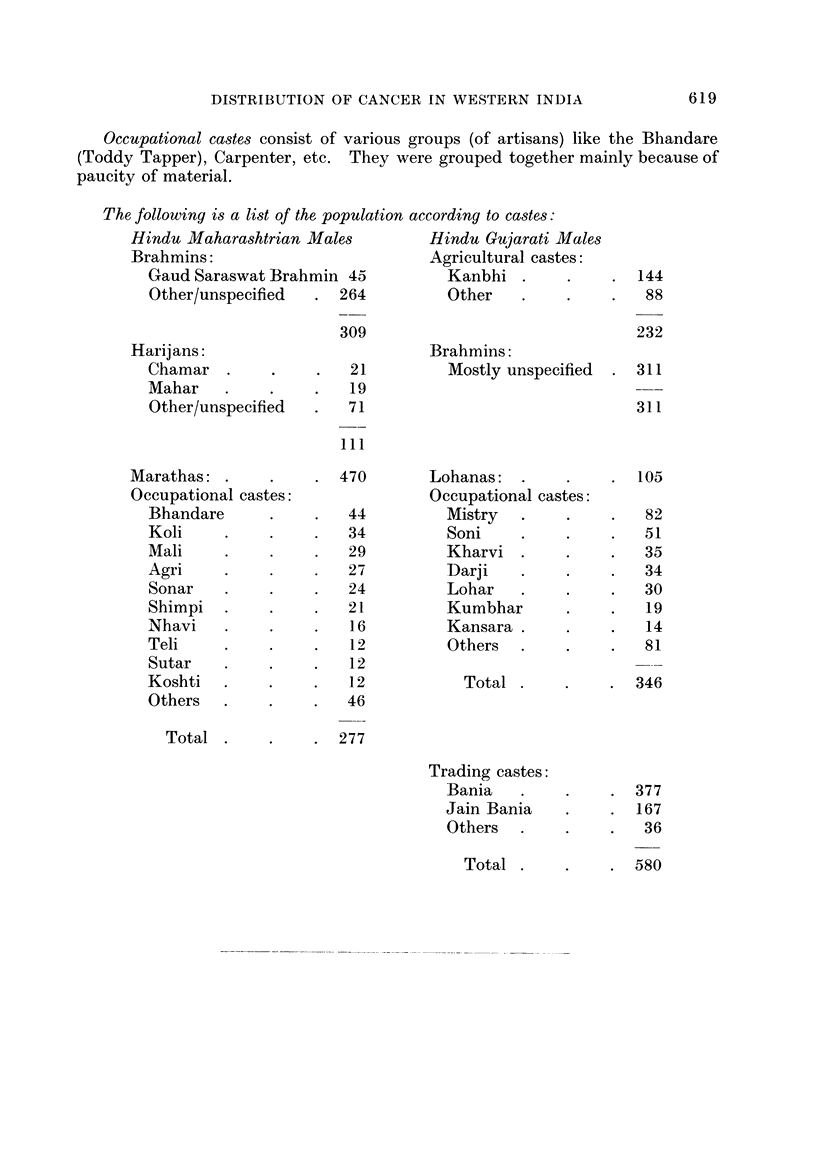


## References

[OCR_00765] Cohen S. M., Waxman S. (1967). Myasthenia gravis, chronic lymphocytic leukemia, and autoimmune hemolytic anemia. "A spectrum of thymic abnormalities?. Arch Intern Med.

[OCR_00771] Goldenberg G. J., Paraskevas F., Israels L. G. (1969). The association of rheumatoid arthritis with plasma cell and lymphocytic neoplasms.. Arthritis Rheum.

[OCR_00793] Miller D. G. (1967). The association of immune disease and malignant lymphoma.. Ann Intern Med.

[OCR_00889] PAYMASTER J. C. (1962). Some observations on oral and pharyngeal carcinomas in the State of Bombay.. Cancer.

[OCR_00806] Papatestas A. E., Alpert L. I., Osserman K. E., Osserman R. S., Kark A. E. (1971). Studies in myasthenia gravis: effects of thymectomy. Results on 185 patients with nonthymomatous and thymomatous myasthenia gravis, 1941-1969.. Am J Med.

[OCR_00895] Paymaster J. C., Sanghvi L. D., Gangadharan P. (1968). Cancer in the gastrointestinal tract in western India. Epidemiologic study.. Cancer.

[OCR_00815] Perlo V. P., Arnason B., Poskanzer D., Castleman B., Schwab R. S., Osserman K. E., Papatestis A., Alpert L., Kark A. (1971). The role of thymectomy in the treatment of myasthenia gravis.. Ann N Y Acad Sci.

[OCR_00901] SANGHVI L. D., RAO K. C., KHANOLKAR V. R. (1955). Smoking and chewing of tobacco in relation to cancer of the upper alimentary tract.. Br Med J.

[OCR_00820] SIMPSON J. A. (1958). An evaluation of thymectomy in myasthenia gravis.. Brain.

[OCR_00822] Souadjian J. V., Silverstein M. N., Titus J. L. (1968). Thymoma and cancer.. Cancer.

[OCR_00903] WYNDER E. L., BROSS I. J. (1961). A study of etiological factors in cancer of the esophagus.. Cancer.

[OCR_00836] Wolf S. M., Rowland L. P., Schotland D. L., McKinney A. S., Hoefer P. F., Aranow H. (1966). Myasthenia as an autoimmune disease: clinical aspects.. Ann N Y Acad Sci.

[OCR_00840] Yunis E. J., Martinez C., Smith J., Stutman O., Good R. A. (1969). Spontaneous mammary adenocarcinoma in mice: influence of thymectomy and reconstitution with thymus grafts or spleen cells.. Cancer Res.

